# Understandability and Actionability of Available Video Information on YouTube Regarding Hemophilia: A Cross-Sectional Study

**DOI:** 10.7759/cureus.29866

**Published:** 2022-10-03

**Authors:** IS Chaitanya Kumar, I Muni Srikanth, Anand Bodade, Amol Khade, Cheranjeevi Jayam, TVN Sriranjitha, Anila Mani

**Affiliations:** 1 Department of Transfusion Medicine and Hemotherapy, All India Institute of Medical Sciences, Mangalagiri, IND; 2 Department of Orthopedics, All India Institute of Medical Sciences, Mangalagiri, IND; 3 Department of Physical Medicine and Rehabilitation, All India Institute of Medical Sciences, Mangalagiri, IND; 4 Department of Dentistry, All India Institute of Medical Sciences, Mangalagiri, IND; 5 Department of Transfusion Medicine, Siddhartha Medical College, Vijayawada, IND

**Keywords:** videos, youtube, pemat score, actionability, understandability, hemophilia

## Abstract

Introduction

With the advent of revolutionary information technology, most general medical information can be accessed by the community at large. However, the factual nature of information, its understandability, and actionability of diseases like Hemophilia are unknown to the general population. Hence the present study has been envisaged to assess the understandability and actionability of available video information on YouTube about Hemophilia.

Methods

A cross-sectional study was performed using the Patient Education Materials Assessment Tool for Audiovisual materials (PEMAT-AV) to assess the understandability and actionability of 50 videos shown by order of relevance utilizing three independent assessors. An online google survey was prepared using the PEMAT questionnaire as a basis and results were recorded and saved as a Microsoft Excel sheet for analysis. Data was analyzed using either Microsoft Excel or an online calculator as the case may be.

Results

A total of 50 short videos on Hemophilia were assessed by three independent assessors using PEMAT. The data so obtained was rechecked by an independent reviewer before data analysis. Three videos were excluded due to non-English language while only two videos out of 50 showed 100% average understandability and actionability. Average understandability and actionability scores range between 34 to 100 percent and 11.1 to 100 percent, respectively. Most videos have higher average understandability than actionability (P value=0.003).

Conclusion

Our study shows there are only a few high-quality short videos available as audio-visual patient education materials on YouTube about Hemophilia. There is a great need to develop content that is beneficial to patients as patient educational material.

## Introduction

The advent of the computer and mobile revolution brought with it the high-speed internet at the present at cheap affordable prices for all. This has increased health education information access to the public through websites, self-help groups, discussion groups, patient groups, chat groups, and app groups [1,2]. However, the information presented available through the internet is only as good as the knowledge of the presenter who can be doctors, paramedical staff, and sometimes patients and other public for generation of awareness. This may cause the presentation of nonactionable, false, or less understandable information to the patient leading to possible increased mortality and morbidity. With the rise of social media, the public is more and more into learning and searching for diseases on free video sites like YouTube.

YouTube and search engines have become the source of information for patients replacing the medical personnel in this time of the 5th Generation advanced cellular technologies and information age. Before a patient approaches a doctor for treatment, many a time they might have approached search engines and are more knowledgeable than we might perceive in the aspect of disease pathogenesis, and management. Hemophilia is an age-old, mostly congenital disease with a lot of information available on the internet and there is a huge chunk of information pertaining to the disease incidence, clinical features, pathogenesis, and management. However, it is difficult to go through all the existing data. To assess how useful the information is for patients, we performed this study to assess the understandability and actionability of videos available on YouTube regarding Hemophilia in terms of understandability and actionability using the Patient Education Materials Assessment tool-Audio/Visual (PEMAT-A/V) [3].

## Materials and methods

The YouTube search engine was searched with the keyword “Hemophilia” sorted by relevance with filters with a duration of under 4 minutes on 5th January 2022 and a playlist was created. We assumed the playlist generated by relevance will be the most seen videos in view of its default nature and given the short duration as most people find themselves reluctant to spend more time to watch lengthy videos when trying to learn something.

A sample size of 30 was assumed sufficient to maintain the integrity of the study and enough to warrant assertions against the present study findings. Assuming exclusion of some videos due to exclusion criteria (non-English videos), additional 20 videos were included taking the total number of videos to 50. The playlist was created using the first 50 videos assuming that the patients will not have the patience to see a long list of videos. The videos in the English language were included in the study out of the 50 videos, thus eliminating three videos. All 47 videos were checked and scored by three independent assessors who have been trained to score in a questionnaire based on the Patient Education Materials Assessment Tool-Audio Visual (PEMAT-AV) using google forms. All three assessors were from the medical background and can understand the intricacies involved in Hemophilia and were to assess the videos with an empathic view towards patients with not much knowledge of Hemophilia. The videos were assessed over a period of three months, values reviewed, videos rechecked for the correctness of data entered, data reviewed, analyzed over a period of six months in the year 2022 as per fixed timelines assessed during the study design. The present study has not been designed to assess the difference between the length of videos and understandability, actionability of videos but to assess the actionability and understandability of short videos alone.

The understandability was to be assessed for content, word choice and style, organization, layout and design, use of visual aids by a 13-point scale. Actionability was directly assessed by the 4-point system. The PEMAT(A/V) system was used in view of its specificity and individual scoring system. A training was provided to three assessors and one reviewer individually. This was followed by the scoring of the 47 videos individually using a google survey sheet for the collection of responses.

A total of 141 responses have been recorded with 47 responses from each of the three assessors. The assessors were made to score the responses independently at different locations to eliminate bias. The recorded data has been coded in such a way as to hide the identity of the assessor by giving a code (A, B, and C) and to not trace the response to a particular assessor except for the principal investigator. The final data has been reviewed by an independent reviewer to check for completeness of responses and to recheck the correctness of scores. The assessor's and reviewer's names have not been disclosed to either of them. After ascertaining the completion of the sheet, the data was analyzed using Microsoft® Excel for computation of actionability and understandability scores of individual assessors and average scores. The average understandability and actionability scores were to be compared using Wilcoxon signed rank test to identify if there is a difference in the mean between the two groups using an online calculator [4].

## Results

**Figure 1:**
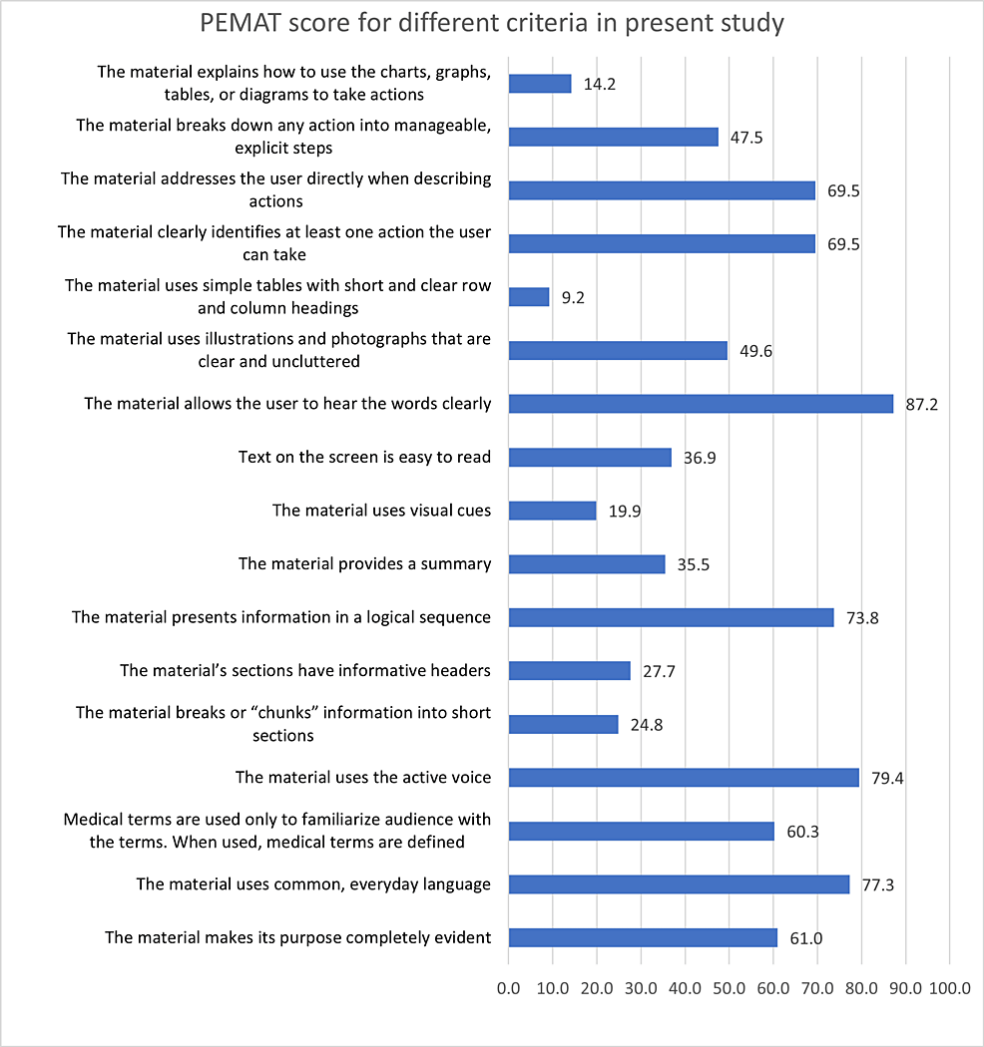
PEMAT Score for different criteria of assessment

The title of the video, creator of content, and the year of upload have been recorded by the assessors (Table 1). The assessor’s responses for each of the videos pertaining to the PEMAT score (Table 2) were incorporated and collected into google survey sheets and the same was analyzed. The cumulative responses of the assessors for each of the 13 understandability and four actionability scoring items have been analyzed (Figure 1).

**Table 1:** The characteristics of videos assessed NA = Not available

S.No	Title of the video	Creator	Year of upload
1	2019 Clinical Research Forum Top Ten | Hemophilia B Gene Therapy	Clinical Research Forum	2019
2	Four facts about being a Hemophilia Carrier	Comprehensive Bleeding Disorders Center	2014
3	A Personal Connection to Hemophilia	BioMarin	2021
4	Anders' Story | Raising a Child With Hemophilia	ROCHE	2020
5	ASK THE EXPERTS- DIAGNOSIS AND MANAGEMENT OF HEMOPHILIA	Foundation for Women & Girls With Blood Disorders	2019
6	Dental management of patients with Hemophilia - Dr. Aniruddha KB	Doctors' Circle - World's Largest Health Platform	2017
7	Diagnosing Hemophilia	Bayer Global	2015
8	Donation of Clotting Factor for Hemophilia Treatment	BIOGEN	2014
9	Dr. Paula James Hemophilia	KGH research institute	2019
10	Emicizumab Prophylaxis in Hemophilia A	NEJM Group	2018
11	Factor Treatment in Hemophilia A and B	Bleeding Disorder Community	2017
12	Female Carriers of Hemophilia May Have Bleeding Symptoms	CSL Behring	2021
13	Free Treatment for Hemophilia & Thalassemia Patients | Conducted by Red Cross | at Eluru	ETV Andhra Pradesh	2018
14	Gene therapy as a future treatment for hemophilia	VJ HemoOnc	2021
15	Gene Therapy for Hemophilia A	Check Rare	2019
16	Hemophilia 5 - Understanding Inhibitors	AboutKidsHealth	2015
17	Hemophilia A and B	Dr. G Bhanu Prakash Animated Medical Videos	NA
18	Hemophilia A Treatment Options	Checkrare	2019
19	Hemophilia Alliance: The Original Managed Care Plan	Checkrare	2019
20	Hemophilia and Gene Therapy	ASGCT	2019
21	Hemophilia clotting cascade- how does your body stop bleeding?	AboutKidsHealth	2021
22	Hemophilia Federation (India) Vignette	Hemophilia Federation India	2019
23	Hemophilia Gene Therapy - One-Off Cure Replacing Factor Injections	Andriy Nemirov	2020
24	Hemophilia Gene Therapy: Expectations for Reliability and Accessibility	IntlSocThrombHemo	2021
25	Hemophilia Overview	CheckRare	2019
26	Hemophilia Patient Stories: José	BioMarin	2021
27	Hemophilia Patient Stories: Mosi	BioMarin	2021
28	Hemophilia royal family (x linked disease example)	MooMooMath & Science	2018
29	Hemophilia Treatment Center: The Bradys' Story | Cincinnati Children's	Cincinnati Children	2019
30	Hemophilia: Keeping the Motivation | Cincinnati Children's	Cincinnati Children	2019
31	Improving Outcomes in Hemophilia A	NEJMVIDEO	2018
32	Joint Damage Caused by Hemophilia | Most Common Complication of Hemophilia	Biotech Review	2018
33	Learning How to Live with Hemophilia	PFIZER	2018
34	Pfizer.com Hemophilia Feature	PFIZER	2016
35	Playing it safe with hemophilia	CDC	2012
36	Sex Linked Traits: Baldness and Hemophilia	BOGOBIOLOGY	2017
37	Starting the Conversation: Hemophilia	CDC	2012
38	Takeda Singapore Plant - making a difference for hemophilia patients around the world	Takeda	2020
39	Tell Me a Story: Youngest Hemophilia Patient to Self-Infuse Grows Up	Cincinnati Children	2012
40	Treating Hemophilia A	NEJM Video	2017
41	Understanding Hemophilia	CAST Pharma	2015
42	Understanding Hemophilia an Inherited Bleeding Disorder and its Types, Causes, and Treatment	Yashoda Hospital	2019
43	Unveiling Hemophilia A | Episode 1: The coagulation team	Roche	2019
44	Unveiling Hemophilia A | Episode 2: Introducing Inhibitors	Roche	2019
45	What is HEMOPHILIA B? What does HEMOPHILIA B mean? HEMOPHILIA B meaning, definition & explanation	The Audiopedia	2017
46	What is hemophilia?	CSL Behring	2017
47	What is the life expectancy in children suffering with Hemophilia? - Dr. G R Subhash K Reddy	Doctors' Circle - World's Largest Health Platform	2016

**Table 2:** PEMAT assessment variables A/V = Audiovisual

S.No	Nature	Type	Scoring variable
1	Understandability	Content	The material makes its purpose completely evident
2		Word Choice and style	The material uses common, everyday language
3			Medical terms are used only to familiarize the audience with the terms. When used, medical terms are defined.
4			The material uses the active voice
5		Organization	The material breaks or “chunks” information into short sections
6			The material’s sections have informative headers
7			The material presents information in a logical sequence
8			The material provides a summary
9		Layout and design	The material uses visual cues (e.g., arrows, boxes, bullets, bold, larger font, highlighting) to draw attention to key points
10			Text on the screen is easy to read (A/V).
11			The material allows the user to hear the words clearly
12		Use of Visual Aids	The material uses illustrations and photographs that are clear and uncluttered
13			The material uses simple tables with short and clear row and column headings
14	Actionability	Actionability	The material clearly identifies at least one action the user can take
15			The material addresses the user directly when describing actions
16			The material breaks down any action into manageable, explicit steps
17			The material explains how to use the charts, graphs, tables, or diagrams to take actions

**Figure 2:**
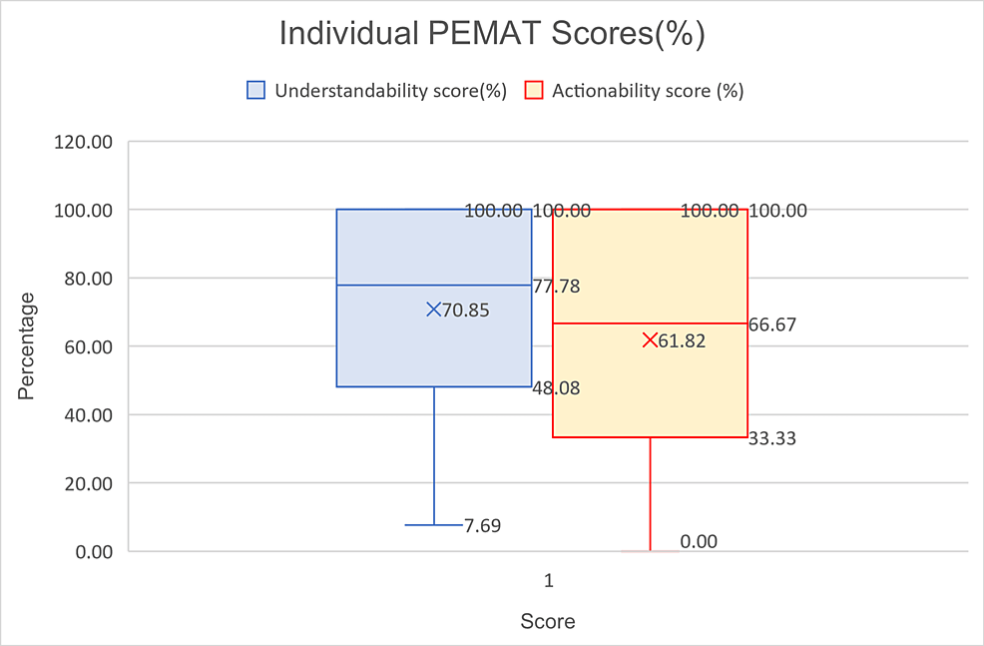
Range of individual PEMAT score (%) for all the videos

The individual PEMAT scores from three assessors for all videos have been analyzed and combinedly ranged from 7.69% to 100% with a median of 77.78% and an inter-quartile range of 48.07% to 100%. The actionability scores similarly ranged between 0% to 100% with a median of 66.67% and an inter-quartile range of 33.33% to 100% (Figure 2). The PEMAT score is by itself subjective, however, it has been tried to mitigate the subjective nature using individual PEMAT scores given by three independent assessors to calculate the average PEMAT scores.

**Figure 3:**
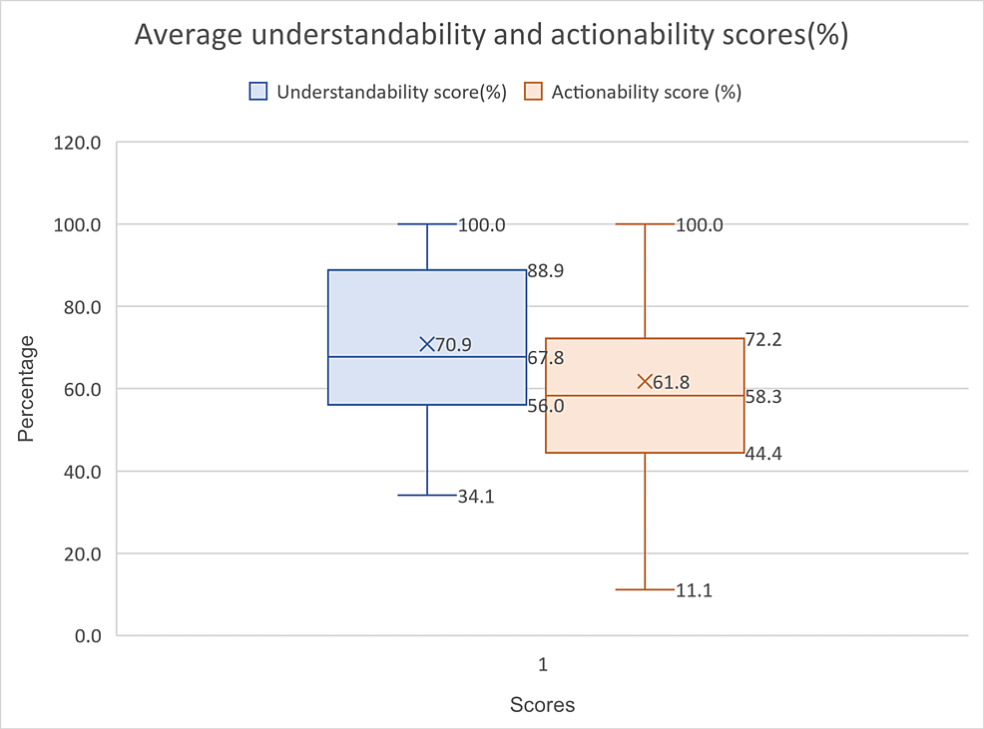
Range of average PEMAT Score (%) for the videos assessed

**Figure 4:**
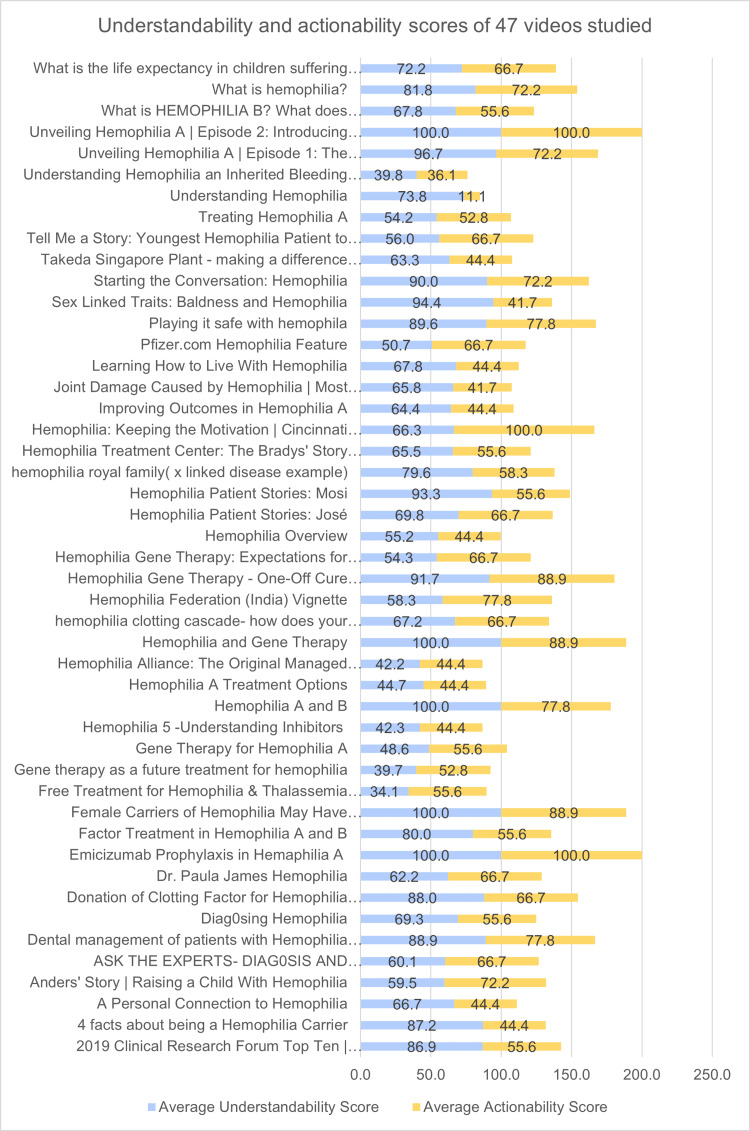
Average understandability and actionability scores of videos assessed

Average understandability and actionability scores ranged between 34 to 100 percent and 11.1 to 100 percent respectively as depicted in Figure 3. The average understandability and actionability of all 47 videos have been analyzed (Figure 4). A total of two videos had 100% understandability and actionability from all three independent assessors.

A Wilcoxon signed rank test was performed to identify the significance of the difference of means between average understandability and actionability using an online calculator and a statistically significant difference was found with a two-sided P value of 0.003 at 0.05 significance levels with a Z value of -2.9743 and W value of 254.

## Discussion

YouTube is the second most visited website after Google and the second most visited social media platform and the world’s first website with audio-visual content [5,6].

Hemophilia is a disease characterized by a congenital or rarely acquired deficiency of coagulation factors, Factors VIII, IX, and/or XI and this leads to spontaneous bleeding or uncontrolled traumatic bleeding due to a defect in the coagulation.

The present study has provided sufficient data to suggest that the existing video material on Hemophilia on YouTube is understandable and actionable. Based on the present study, which required 50 videos to be assessed with a final result of only two 100% understandable and actionable videos, it is presumed that at least 25 short videos must be seen to get at least one 100% understandable and 100% actionable video by a patient. In the present study, there are few videos with zero actionability scores which indicates that the video content developers are more into providing information to the patients than providing information as to what to be done next.

In the present study, out of 47 videos screened, seven and 13 videos had average understandability and actionability scores of less than 50% respectively. The mean understandability score (70.9%) has just crossed the threshold of the PEMAT score for patient education materials of 70% [3], while the mean actionability score is below a threshold level (61.8%).

No literature could be found that searched the video material on hemophilia to assess understandability and actionability to date. There are certain studies which have assessed understandability and actionability using PEMAT scores but on different diseases like diabetes [7], cardiovascular disease risk [8], asthma [9], macular degeneration [10], sinusitis [11], vocal cord paralysis [12], cardiac electrophysiological procedures [13], shoulder arthroscopy [14], over the counter rapid antigen COVID-19 testing [15], fall prevention [16], etc. Most of these studies have higher understandability scores and poor actionability studies as has been identified in our study also. One finding that can be observed is the earlier videos in studies that were more emphasizing understandability while the recent videos and recent studies have increased actionability terms incorporated into the videos. This can improve further as the content developers get to know how to create content useful for patients.

The present study is limited by the sample size and study design to assess the understandability and actionability of short videos on Hemophilia only. The sample size could not be calculated in view of the paucity of data to use in sample size calculation. However, the present data can be utilized in designing better studies with a larger sample size in the future with more varied objectives.

## Conclusions

Our study shows there are only a few high-quality short videos available as audio-visual patient education materials on YouTube about Hemophilia. Most of the short videos are understandable but poorly actionable. There is a great need to develop content that is beneficial to patients as patient educational material.
